# The impact of COVID-19 on clinical practice and well-being of global mental health professionals

**DOI:** 10.1192/j.eurpsy.2021.105

**Published:** 2021-08-13

**Authors:** T. Rebello, G. Reed

**Affiliations:** 1 Psychiatry, Columbia University, New York City, United States of America; 2 Psychiatry, Columbia University Vagelos College of Physicians and Surgeons, New York, United States of America

**Keywords:** COVID-19, burnout, telehealth, mental health services

## Abstract

Some of the most direct and brutal effects of the COVID-19 pandemic are experienced by health care professionals who are working in demanding environments while having to deal with their own fears of infection and mortality. To assess the impact of COVID-19 on the practice and well-being of global mental health professionals, we designed a three-part, longitudinal, internet-based study. Here we present data from part 1, implemented in June-July 2020 in six languages to members of WHO’s Global Clinical Practice Network composed of 15,500 mental health practitioners. The study assessed COVID-19’s impact on: work circumstances; occupational well-being; use and transition to telehealth; and expectations, needs and recommendations. 2,505 mental health professionals from 126 countries responded to the study (47% psychiatrists). 93.7% of respondents were currently practicing and 70.9% continued to see patients in person. The impact on clinical workload varied in terms of direction and extent depending on type of service provided and country of practice. Most participants had started or increased their use of telehealth services, and we identified a need for training to support telehealth use. Overall, clinicians scored high on well-being indices. However, a subset scored above the cutoff for low well-being and reported a significant number of post-traumatic symptoms. Five factors affected work-related stress: fear of infection, severe COVID-related events, life disruption, lack of adequate protection and role disruption. Data from this study will provide information relevant for the design, development, and integration of mental health services in the continuing pandemic, and in similar future scenarios.

**Disclosure:**

No significant relationships.

